# Essay Read before the Military Tract Medical Association

**Published:** 1873-12-01

**Authors:** Henry B. Upton

**Affiliations:** Osceola, Ill.


### ESSAY READ BEFORE THE MILITARY TRACT MEDICAL ASSOCIATION.

BY HENRY B. UPTON, M.D., OSCEOLA, ILL.

*Mr. President and Gentlemen:*

The effort to advance medical science by experimental practice brings to our notice some new remedies every year; and yet disease and death, unmindful of their claimed potent influence, march on unchecked. Could we, as a profession, supply with our powders, pills, solutions, and decoctions, nerve-force as readily as the engineer gives power to his machine, we would be most fortunate indeed.

I am frequently inclined to think that we have *too much materia medica*, and *too little common sense.* I am fully convinced that many of the substances held in high repute by medical professors, and upon which we have often relied as possessing wonderful remedial power, are almost if not totally inert, so far as any action upon the functions of life are concerned. I have treated cases where ordinary doses of opiates were powerless, but bread pills and small doses of aquæ pura, administered with punctilious regularity, were all-powerful and magical in their action.

While I would not condemn at one sweep the therapeutics of our accepted materia medica, I would most earnestly caution against the too implicit trust sometimes held in their power. The most powerful medicines are the most poisonous, and should be most carefully applied. Meddlesome application of materia medica is as bad as meddlesome midwifery; and the way some of the victims of the profession have been stuffed with villainous compounds, and abluted with multitudinous mixtures — oleaginous, alcoholic, alkaline and aquæous (frequently the quadruple combination) — has been sufficient to carry many to their last long home, where, if they wake to consciousness, they will hope no doctors may come. Still, many recover in spite of wholesale medication—spared monuments, not of the doctor's skill, but of the great recuperative power of nature. The very fact that different members of this Association treat the same abnormal conditions of the human body with such a diversity of remedies, often absolutely opposite in their action, utters volumes in denunciation of any course of practice trusting implicitly upon the virtue in our materia medica. It is more than possible that those who recover would do so without the aid of a physician, although it might not be in a strictly scientific manner. I have three cases to report, where the foregoing remarks are illustrated.

Case I.—Was called to visit a man two miles from my office. The messenger clamored for haste on my part, and I was soon in presence of the patient. He was rolling upon the bed in agony, groaning and shrieking; was, as he expressed it, "dreadful sick at the stomach, and burning up." Found, upon examination, a rapid pulse, and the extremity of the tongue red and raw. The attendants, anxious to *do something*, had been giving him liberal doses of some patent pain-killer, which he described as being "hot as hell." I immediately ordered ice, and directed him to partake of small pieces, and swallow one every three or five minutes, and quietly sat down by the fire to warm and await the result. After eating three or four pieces, he anxiously inquired if I was not going to give him some medicine. I told him to eat a few pieces of ice first. In fifteen minutes he was easier; in twenty-five almost free from pain. With occasional use of ice through the night, when the burning sensation was felt, and a few directions about diet, he immediately recovered, much to the astonishment of the old women, who said, "That there is the fust time the pain-killer did n't cure the colic!"

Case II.—A messenger came in haste, stating that Mr. T—— was very sick, and that old Dr.—— (a quack) had worked at him several hours, and could not relieve him. I at first refused to attend, as I would not counsel with Dr. ——; but upon being assured that he left just as I was sent for, I hastened to fill the call. Found the patient in great suffering; complained of severe pain in the right inguinal region. I discovered a slight hardness, unnatural to the part, by touch, and diagnosed the case typhlitis. When I arrived there were two persons very laboriously rubbing his arms with ammoniacal liniment. Of course I discontinued that, as it would be as useless as to apply a sinapism to the pedal extremity for epistaxis. I applied a huge suppository of opium, thus endeavoring to relieve pain; ordered hot cloths to the part, often changed, and commenced giving cathartics. All my efforts proved useless; and I concluded to try a purely mechanical relief, for I considered the cause of the inflammation to be impacted fæces. I prepared a quantity of fluid for an enema, using castor oil, soap, salt and water, and nearly three hours I injected with all the force I could get from one of Richardson's syringes. He would declare I was filling him full clear up, and then he would get upon the stool. As soon as he had finished, I would commence again. I knew I must force the fluid to the cœcum, or it would do no good. I was rewarded with complete success before three hours, all the harm done to the patient being to leave him very weak and exhausted. A generous diet brought him around in three days perfectly cured, and—abundantly able to grumble about the doctor's bill!

Case III.—This is a case instructive only in point of mental action. Was called to see a lady who, having some slight visceral derangement, had a tendency to hypochondriasis. Insomnia being the most marked symptom, I prescribed the usual articles in such cases, without remark. They produced no effect whatever, as she informed me the following day. Taking the matter into careful consideration, I concluded to try an experiment. I prepared some good-sized bread pills, and filled a two ounce vial with water, changing the taste and color with sweet spirits of nitre and tincture of gentian. Armed with these, I called, towards night, and informed her that I had thought carefully upon her case, and had now fixed a medicine which would cause her to sleep sweetly. I enjoined the utmost regularity in the taking of the preparations—first a pill, then in one hour a teaspoonful from the bottle; again in one hour a pill, and so on. I told her that after taking a pill and a teaspoonful a sleepy feeling would be apparent; her eyelids would feel heavy; in one more hour she would feel quite sleepy; but she must not give up to it, but after the next dose she could, and might sleep; and she probably would not awake until morning, unless her bowels should need attention; and I advised that she should make all necessary evacuations the last hour, so that she might rest undisturbed. I left, and upon calling the next day found everything had proceeded as I had set forth. She recovered without further medication, firmly in the belief that I had "hit her case exactly."

I wish to briefly call attention to some of the new remedies, more to elicit experience than to offer any settled views. Gelsemium: The use of, in my practice, unsatisfactory, failing in most cases to obtain the effect claimed by its supporters. Chloral hydrate: Have used it with some success, and find it very useful where I wish to administer a hypnotic with some degree of stimulation. Have found it a valuable remedy with other appropriate treatment in gastric fever. In many cases I have not been pleased with its action; and, although a valuable agent, it can never meet the expectations of its most ardent friends. Sulphite of soda: I have used it with considerable success as an anti-ferment in cases of dyspepsia arising from fermentation. Have found it necessary often to combine with antacids. It is highly recommended by some in all zymotic diseases. I have never given it a trial in that direction. Xylol: This is being used in some places in smallpox, with marked success. It is administered in ten to fifteen drop doses, four times a day, given in wine, syrup or water.

